# Verrucous carcinoma: a difficult differential diagnosis

**DOI:** 10.11604/pamj.2024.47.118.37715

**Published:** 2024-03-13

**Authors:** Brendan Guegan

**Affiliations:** 1 Department of Oral Surgery, Nimes University Hospital, Nimes, France

**Keywords:** Verrucous carcinoma, verrous hyperplasia, proliferative verrucous leukoplasia

## Image in medicine

A woman aged 85 years old was followed up for a giant verrucous carcinoma of the oral cavity. Looking at the patient´s history, P16 was not over-expressed, Ki67 was expressed in basal layers, and no HPV was detected. Significant squamous hyperplasia of verrucous architecture with parakeratosis. Ulcerated areas are present on the surface. The chorion is the site of a moderate lymphocytic and plasma cell inflammatory infiltrate associated with numerous neutrophils in intraepithelial exocytosis with the formation of superficial micro-abscesses. Treatment with external radiotherapy, with an incomplete response rate, is to be completed with surgical removal. Difficult diagnosis between verrucous hyperplasia, proliferative verrucous leukoplasia and verrucous carcinoma. The essential rule in this type of lesion is the importance of deep and multiple biopsies, to rule out conventional squamous cell carcinoma and to distinguish verrucous carcinoma from verrucous hyperplasia and proliferative verrucous leukoplasia.

**Figure 1:**
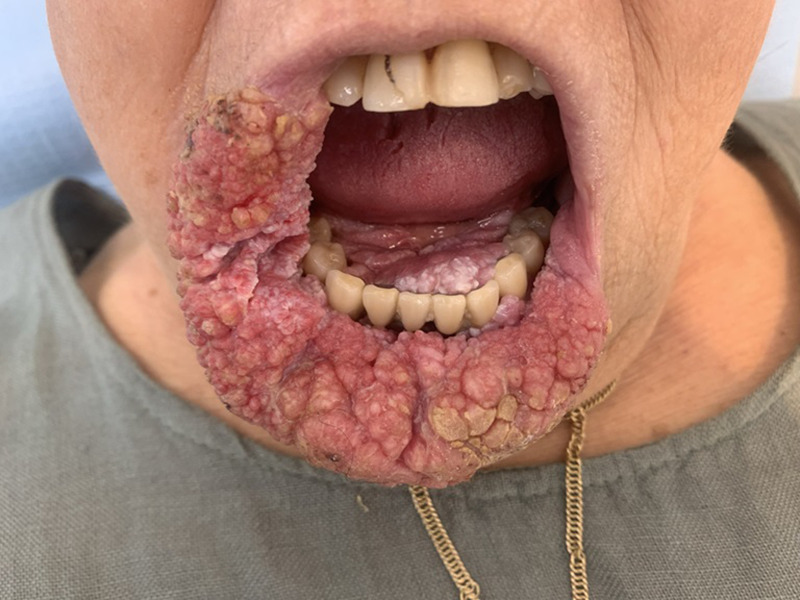
verrucous carcinoma

